# Ruthenium-based electrocatalyst for efficient acidic water oxidation in PEM water electrolysis for H_2_ production

**DOI:** 10.1039/d5ra05145b

**Published:** 2025-09-23

**Authors:** Yinan Tao, Ruilin Zhang, Junyan Chen, Wubin Weng, Yong He, Zhihua Wang

**Affiliations:** a State Key Laboratory of Clean Energy Utilization, Zhejiang University Hangzhou 310027 P. R. China wangzh@zju.edu.cn; b Qingshanhu Energy Research Center, Zhejiang University Hangzhou 311300 P. R. China; c Hoymiles Power Electronics Inc. Hangzhou 310015 P. R. China

## Abstract

Proton exchange membrane (PEM) water electrolysis is a promising and sustainable technology for hydrogen production. Currently, the anode catalysts used in PEM water electrolysis are predominantly iridium-based electrocatalysts, which are extremely precious and scarce. In this study, we report a mixed (Ru–W)O_*x*_ catalyst as a promising alternative to iridium-based catalysts. The (Ru–W)O_*x*_ catalyst was synthesized using a simple Pechini–Adams method, and its microstructure and electrochemical performance were optimized by controlling the Ru/W doping ratio and the synthesis temperature. Among the synthesized catalysts, the Ru_6_W_4_O_*x*_ catalyst prepared at 400 °C demonstrated the best oxygen evolution reaction (OER) activity and stability, achieving an overpotential of only 140.32 mV at 10 mA cm^−2^. Furthermore, after a 150 hours stability test, no significant loss in catalytic activity was observed. When applied to the anode of a PEM water electrolyzer, the Ru_6_W_4_O_*x*_-400 °C catalyst exhibited an impressively low cell voltage of 1.784 V at 2 A cm^−2^. The energy consumption is as low as 4.34 kWh m^−3^ H_2_. These results provide new insights for developing efficient and stable non-iridium-based OER catalysts for PEM water electrolysis.

## Abstract 2

The developed Ru_6_W_4_O_*x*_ showed exceptional OER activity with an overpotential of 140.32 mV at 10 mA cm^−2^, far superior to commercial RuO_2_. A PEMWE using Ru_6_W_4_O_*x*_ achieved 1.784 V at 2 A cm^−2^, 4.34 kWh m^−3^ H_2_ and an electrolysis efficiency of 81.72%.

## Graphical abstract

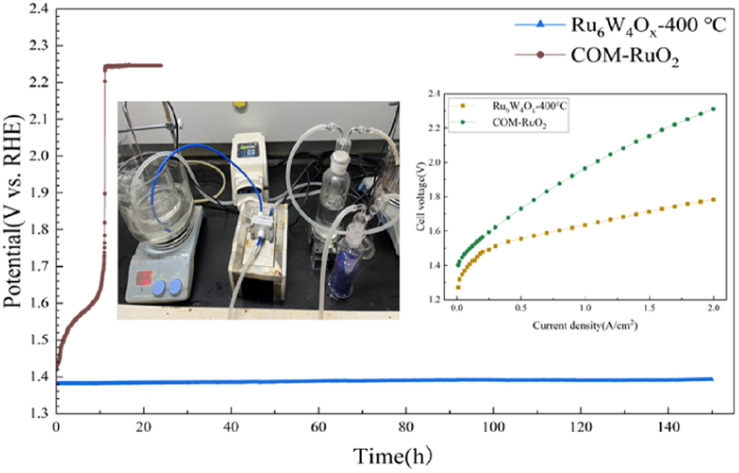

## 1 Introduction

Hydrogen is considered a crucial element for achieving deep decarbonization of modern energy systems. Water electrolysis powered by renewable electricity offers a “zero-carbon” pathway for hydrogen production. With the large-scale expansion and cost reduction of renewable energy, the green hydrogen production pathway has become highly promising. Among various water electrolysis technologies, proton exchange membrane (PEM) electrolysis stands out due to its fast dynamic response, wide operating load range, and compact system structure.^1–4^ These features make PEM electrolysis suitable for coupling with intermittent renewable energy sources to produce green hydrogen.

PEM electrolysis technology uses a sulfonated polymer membrane as the electrolyte, with hydrogen ions(H^+^) as the ionic carriers. Deionized water permeates through the proton-conducting membrane, enabling electrochemical reactions that consist of two half-cell processes:^5^

Anode – Oxygen Evolution Reaction (OER):

1 H_2_O → 2H^+^ + 1/2O_2_ + 2e^−^, *U*_0_ = 1.23 V

Cathode – Hydrogen Evolution Reaction (HER):

2 2H^+^ +2e^−^ → H_2_, *U*_0_ = 0 V

At the anode, water is split into oxygen molecules, protons, and electrons. This process imposes harsh working conditions on the components of PEM electrolyzers, including high potential, oxidative stress, and severe corrosiveness.^6^ These stringent conditions significantly limit the choice of materials. Currently, OER catalysts suitable for PEM systems still rely on the extremely scarce and expensive precious metal iridium (Ir) and its oxides. Consequently, extensive research efforts have been devoted to developing electrocatalysts with reduced Ir content while achieving higher activity and stability.^7–10^ Strategies include doping with non-precious metals, incorporating support materials, and tailoring catalyst morphologies.^11–13^

Compared to Ir, ruthenium (Ru) is the most active metal for acidic OER due to its optimal binding energy with oxygen-related intermediates.^14^ Additionally, Ru is more abundant in Earth's crust and significantly less expensive than Ir (Ir ≈ US$140/g, Ru ≈ US$16/g).^15^ However, the stability of Ru-based catalysts remains a critical challenge, as Ru is prone to excessive oxidation under acidic conditions. This leads to lattice oxygen loss, structural instability, and accelerated catalyst degradation. Thus, while Ru exhibits excellent OER activity, improving its structural stability during prolonged operation remains a key hurdle.

In recent years, researchers have focused on using metal doping strategies to enhance the performance of Ru-based catalysts.^15–30^ High-entropy or multi-metal oxides based on Ru can form strong metal–oxygen (M–O) bonds, suppressing lattice oxygen over-oxidation.^8^ For example, Su *et al.* synthesized Cu-doped hollow porous RuO_2_ polyhedral structures using a Cu-BTC precursor. The Cu-doped RuO_2_ exhibited excellent OER performance, achieving a current density of 10 mA cm^−2^ with a low overpotential of 188 mV in 0.5 M H_2_SO_4_. It also demonstrated high stability over 10 000 CV cycles and 8 hours of chronoamperometric testing. The enhanced performance was attributed to the highly unsaturated Ru sites on high-index facets and the electronic structure modulation induced by Cu doping.^30^ Chen *et al.* reported Mn-doped RuO_2_, achieving an overpotential as low as 158 mV at 10 mA cm^−2^ in 0.5 M H_2_SO_4_, with moderate stability. DFT calculations indicated that Mn doping regulated the d-band center of Ru sites, enhancing intrinsic activity.^22^

Studies on Ru and Tungsten (W) co-doped oxides are also emerging. Wang *et al.* synthesized the heterogeneous catalyst W-IrRu_3_O_*x*_. The W-IrRu_3_O_*x*_ catalyst exhibits high corrosion resistance, along with a low overpotential of 249 mV at 10 mA m^−2^ and a low Tafel slope of 64 mV dec^−1^. During the chronoamperometric test, *η*_10_ of W-IrRu_3_O_*x*_ increases from 231 to ∼300 mV in the first ∼30 h and then remains nearly unchanged during the subsequent period.^21^ However, challenges remain, including complex synthesis methods, high precious metal content, and limited testing under high current densities in practical electrolyzers.

In this study, a series of (Ru–W)O_*x*_ catalysts were synthesized using a simple two-step method. By systematically adjusting doping ratios and calcination temperatures, a comprehensive evaluation was conducted for the developed catalysts' performance in OER, including activity, reaction kinetics, and long-term stability. The results will provide important insights into optimizing Ru-based catalysts and preparation parameters, offering new strategies for designing efficient and stable OER catalysts under acidic conditions.

## 2 Experimental methods

### 2.1 Synthesis of catalysts

The (Ru–W)O_*x*_ catalysts were synthesized using the Pechini–Adams method. By controlling the precursor ratios and calcination temperatures, nanoscale catalysts with smaller particle sizes and desirable morphologies were obtained, which enhanced the electrochemically active surface area and improved reaction efficiency.^31^ RuCl_3_·3H_2_O and WCl_6_ with varying molar ratios were dissolved in ultrapure water under vigorous stirring and ultrasonicated for 30 minutes. Separately, 150 mg of EDTA and 60 mg of citric acid were dissolved in ultrapure water and gradually added to the RuCl_3_·3H_2_O and WCl_6_ solution. The mixture was then stirred continuously at room temperature for 4 hours to ensure uniform dispersion of the components.

Subsequently, the mixture was heated in a water bath at 90 °C while stirring until the solution completely evaporated. The dried catalyst was transferred to a ceramic crucible and calcined in a muffle furnace at a temperature of 400 °C for 2 hours, with a ramp rate of 5 °C min^−1^. After being annealed, the samples were allowed to cool naturally to room temperature, followed by multiple rounds of high-speed centrifugation using 10 wt% HClO_4_ solution, ultrapure water, and ethanol to wash the catalyst. The supernatant was discarded after each wash. Finally, the sediment was dried in an oven at 80 °C, collected, and stored in sealed sample vials under controlled temperature and humidity.

The catalysts were labeled based on the molar ratios of Ru to W in the precursors as Ru_9_W_1_O_*x*_-400 °C, Ru_8_W_2_O_*x*_-400 °C, Ru_7_W_3_O_*x*_-400 °C, Ru_6_W_4_O_*x*_-400 °C, Ru_5_W_5_O_*x*_-400 °C, and Ru_4_W_6_O_*x*_-400 °C. Additionally, a single-metal Ru-based oxide catalyst was prepared using a similar method and labeled as RuO_*x*_-400 °C.

### 2.2 Characterization of catalysts

The surface morphology and microstructure of the (Ru–W)O_*x*_ catalysts were characterized using a transmission electron microscope (TEM, FEI Tecnai G2 F20 S-TWIN and FEI Tecnai G2 F20 S-TWIN) equipped with energy-dispersive X-ray spectroscopy (EDX) to analyze elemental distribution on the surface. X-ray diffraction (XRD, X-pert Powder) was employed to determine the structural and crystalline properties of the catalysts, with a scan range of 2*θ* = 20−80°. X-ray photoelectron spectroscopy (XPS, Thermo Scientific K-Alpha) was used to analyze the surface elemental composition and oxidation states of the catalysts.

### 2.3 Electrochemical Measurements

The electrochemical performance of the synthesized (Ru–W)O_*x*_ catalysts and commercial RuO_2_ catalyst (hereafter referred to as COM-RuO_2_, purchased from Shanghai Macklin Co., Ltd) was evaluated using a standard three-electrode electrochemical cell system. The experimental setup included an electrochemical workstation (AUTOLAB PGSTAT302N) and a 100 mL cylindrical glass electrolysis cell. The three-electrode system comprised a 3 mm diameter glassy carbon electrode (working electrode), a 0.5 mm × 37 mm platinum wire (counter electrode), and a HgSO_4_ reference electrode (reference potential of 0.65 V *vs.* RHE). The electrolyte was 0.5 M H_2_SO_4_ solution. For consistency, all potentials were converted to values *versus* reversible hydrogen electrodes according to *E*(RHE) = *E*(WE) + 0.65 + 0.0592 pH.

The catalyst ink was prepared by mixing 2 mg of (Ru–W)O_*x*_ or COM-RuO_2_ catalyst, 20 μL of 5 wt% Nafion solution, and 285 μL of isopropanol. The mixture was ultrasonicated for more than 30 minutes at temperatures below 25 °C. A micropipette was used to deposit 1 μL of the ink onto the black surface of the glassy carbon electrode, allowing each layer to dry completely before adding the next. A total of 5 μL of ink was applied.

Linear sweep voltammetry (LSV) was performed to obtain the polarization curves and corresponding overpotential values, with the potential range set from 1.3 to 1.8 V and a scan rate of 10 mV s^−1^. Electrochemical impedance spectroscopy (EIS) was used to measure the impedance of the catalyst under different frequencies, applying a sinusoidal perturbation of 10 mV at 1.45 V *vs.* RHE, with a frequency range of 10 kHz to 10 mHz. The cost-effectiveness of the catalyst was evaluated by calculating the mass activity(normalized by Ru). The Ru mass fraction in the catalyst was determined based on EDX results obtained from TEM characterization.

Cyclic voltammetry (CV) was performed to determine the double-layer capacitance (*C*_dl_), revealing the electrochemical surface area (ECSA). Scans were conducted within the non-faradaic range of 0.82–0.92 V at scan rates of 20, 40, 60, 80, and 100 mV s^−1^. The ECSA was evaluated from the *C*_dl_ according to ECSA = *C*_dl_/*C*_s_*m*_loading_. *C*_s_ is the sample's specific capacitance or the capacitance of an atomically smooth planar surface of the material per unit under identical electrolyte conditions, and is usually assumed as 0.035 mF cm^−2^ in H_2_SO_4_ electrolyte; *m*_loading_ is the mass of the load catalyst. Chronoamperometry (CA) was used to assess the long-term electrochemical stability of the catalysts by applying a constant current density of 10 mA cm^−2^ to the electrode and recording the potential over a 24 hours period.

## 3 results and discussion

### 3.1 structures and morphologies of (Ru–W)O_*x*_ electrocatalysts

**Fig. 1:**
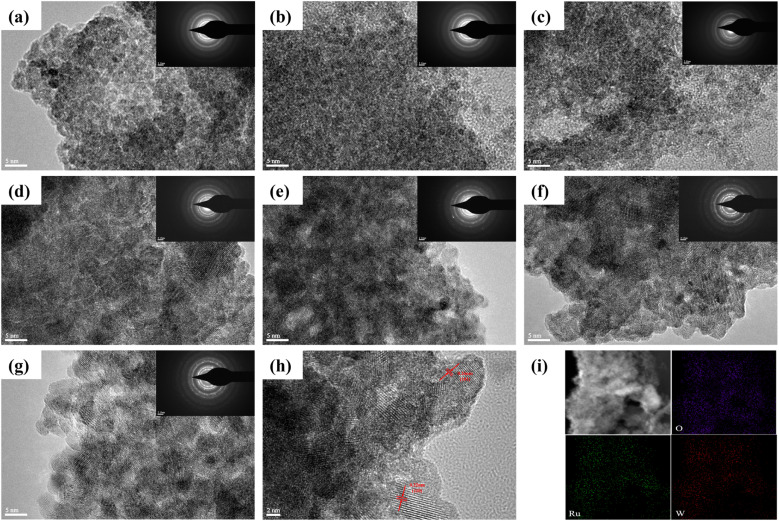
TEM and SEAD images of (Ru–W)O_*x*_ catalysts (a) Ru_4_W_6_O_*x*_-400 °C, (b) Ru_5_W_5_O_*x*_-400 °C, (c) Ru_6_W_4_O_*x*_-400 °C, (d) Ru_7_W_3_O_*x*_-400 °C, (e) Ru_8_W_2_O_*x*_-400 °C, (f) Ru_9_W_1_O_*x*_-400 °C, (g) RuO_*x*_-400 °C, (h) marked HRTEM image of Ru_6_W_4_O_*x*_-400 °C, (i) corresponding EDX elemental mapping of Ru_6_W_4_O_*x*_-400 °C.

The TEM images of catalysts with different Ru and W ratios and their corresponding selected area electron diffraction (SAED) patterns are shown in Fig. 1a–g. The images reveal a clear trend in crystallinity as the Ru content increases. As the Ru content increases and the W content decreases, the particle morphology gradually transitions from irregular amorphous structures to denser and more regular crystalline structures, with an increasing number of well-defined RuO_2_ lattice fringes. Overall, W primarily appears in low-crystallinity or amorphous forms at this temperature, resulting in the presence of dispersed amorphous regions in the overall morphology. High-resolution TEM (HRTEM) analysis indicates well-defined lattice fringes with clear crystal surface spacing of about 3.2 Å (Fig. 1h), corresponding precisely to the (110) crystallographic plane of rutile RuO_2_. This observation confirms the preservation of the host rutile structure in (Ru–W)O_*x*_, consistent with the XRD results. Elemental mapping (Fig. 1i) demonstrates homogeneous spatial distribution of Ru, W, and O at nanoscale, indicating that W is uniformly doped in the RuO_2_ matrix, rather than clustered in localized areas. The EDX analysis results, shown in Table 1, confirm that the Ru/W ratio in the synthesized catalysts is close to the initial feed ratio, indicating the successful synthesis of (Ru–W)O_*x*_ catalysts. These results were subsequently used for the calculation of mass activity and ECSA.

**Table 1:** Initial feeding and final element composition of (Ru–W)O_*x*_ catalysts

Catalysts	Initial feeding			The synthesized catalysts			
	RuCl_3_·3H_2_O (mg)	WCl_6_ (mg)	W/Ru (at%)	Ru (wt%)	W (wt%)	O (wt%)	W/Ru
RuO_*x*_-400 °C	261.47	0	0	77.35	0	22.65	0
Ru_9_W_1_O_*x*_-400 °C	235.32	39.66	0.11	72.67	15.85	11.48	0.12
Ru_8_W_2_O_*x*_-400 °C	209.18	79.11	0.25	53.44	22.94	23.62	0.24
Ru_7_W_3_O_*x*_-400 °C	183.03	118.97	0.43	42.51	35.26	22.23	0.45
Ru_6_W_4_O_*x*_-400 °C	156.88	158.62	0.67	39.88	47.82	12.29	0.67
Ru_5_W_5_O_*x*_-400 °C	130.74	198.28	1	30.22	51.61	18.17	0.93
Ru_4_W_6_O_*x*_-400 °C	104.59	237.94	1.5	25.78	62.35	11.87	1.33

**Fig. 2:**
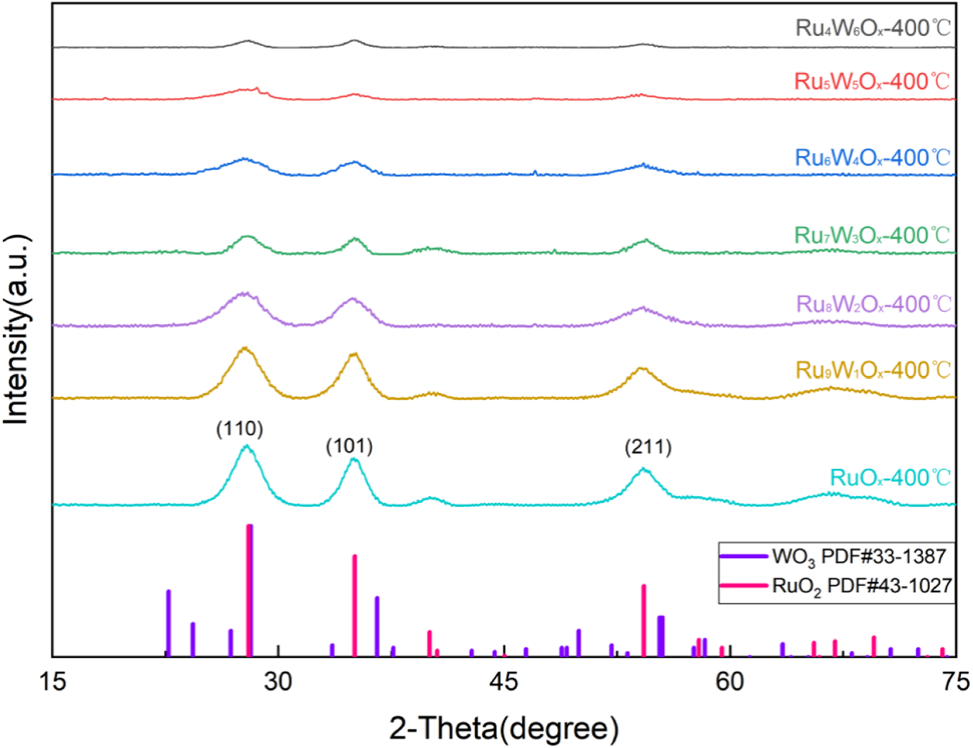
XRD patterns of (Ru–W)O_*x*_ catalysts.

The XRD patterns, shown in Fig. 2, show that all samples exhibit characteristic diffraction peaks corresponding to RuO_2_ (JCPDS#43-1027), with three prominent peaks observed near 28°, 35°, and 54°, which can be attributed to the (110), (101), and (211) planes of RuO_2_, respectively. No distinct peaks corresponding to W oxides were observed, which is consistent with the TEM results. As the W doping ratio increases, the characteristic peaks of RuO_2_ become less prominent, indicating that the addition of W affects the lattice structure of Ru oxides. At lower W doping levels, the diffraction peaks of RuO_2_ remain sharp, while the peaks associated with WO_3_ are relatively weak. This suggests that small amounts of W doping do not significantly disrupt the RuO_2_ crystalline phase but may induce partial substitution or lattice distortion without forming a separate WO_3_ phase. Furthermore, with increasing W content, the characteristic peaks near 28°, 35°, and 54° gradually shift slightly toward higher angles. This shift indicates lattice expansion, which can be attributed to the difference in ionic radii between Ru^4+^ (0.62 Å) and W^4+^ (0.66 Å).

**Fig. 3:**
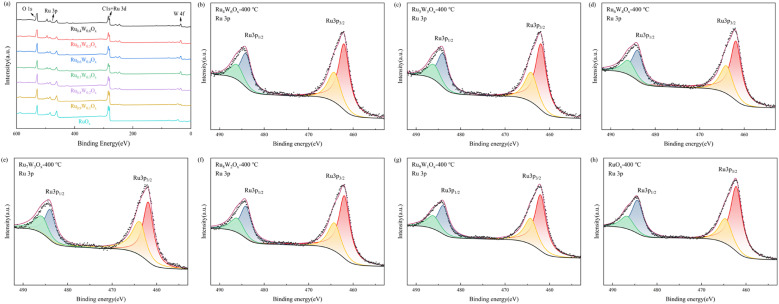
XPS full spectra and Ru 3p spectra of (Ru–W)O_*x*_ catalysts (a) XPS full spectra, (b) Ru_4_W_6_O_*x*_-400 °C, (c) Ru_5_W_5_O_*x*_-400 °C, (d) Ru_6_W_4_O_*x*_-400 °C, (e) Ru_7_W_3_O_*x*_-400 °C, (f) Ru_8_W_2_O_*x*_-400 °C, (g) Ru_9_W_1_O_*x*_-400 °C, (h) RuO_*x*_-400 °C.

Surface chemical states of the catalysts were systematically studied through XPS. The XPS full spectra confirm the presence of W in (Ru–W)O_*x*_, consistent with the elemental mapping results. Since C 1s and Ru 3d spectra overlap, the secondary intense peak of Ru (Ru 3p) was measured for further contrast. The Ru 3p spectra (Fig. 3 and Table 2) could be deconvoluted into two pairs of peaks. The peaks at binding energies of 484–487 (Ru 3p_1/2_) and 461–464 eV (Ru 3p_3/2_) could be attributed to Ru^4+^ and low-valence Ru species. Additional peaks at higher binding energies (463.7–464.7 eV for Ru 3p_3/2_ and 485.6–486.8 eV for Ru 3p_1/2_) corresponded to satellite peaks of Ru. Compared with RuO_*x*_, the Ru 3p peaks of (Ru–W)O_*x*_ were shifted to a lower binding energy. Generally, a decrease in binding energy suggests an increase in electronic density, potentially associated with a lower oxidation state.^32^ With the W doping ratio increasing, the binding energy first decreased and then increased, with Ru_6_W_4_O_*x*_ exhibiting the lowest binding energy. This indicates the lower Ru oxidation state and higher electron cloud density of Ru_6_W_4_O_*x*_, which benefit the adsorption of reactants and electron transfer on the catalyst surface, accelerating the catalytic reaction, thereby enhancing catalytic performance. The trend of Ru 3p binding energy (lowest in Ru_6_W_4_O_*x*_) is consistent with the OER activity trend (lowest overpotential in Ru_6_W_4_O_*x*_, Fig. 6a) and charge transfer resistance (lowest in Ru_6_W_4_O_*x*_, Fig. 6c).

**Table 2:** The binding energy of Ru 3p peaks

Catalyst	Binding energy (eV)			
	Ru 3p_3/2_	Ru 3p_3/2_ sat.	Ru 3p_1/2_	Ru 3p_1/2_ sat.
RuO_*x*_-400 °C	462.17	464.61	484.4	486.73
Ru_9_W_1_O_*x*_-400 °C	462.03	464.22	483.9	486.12
Ru_8_W_2_O_*x*_-400 °C	461.95	464.18	484.1	486.08
Ru_7_W_3_O_*x*_-400 °C	461.90	463.7	483.91	485.64
Ru_6_W_4_O_*x*_-400 °C	461.86	463.99	483.97	486.01
Ru_5_W_5_O_*x*_-400 °C	461.93	464.12	483.99	486.02
Ru_4_W_6_O_*x*_-400 °C	462.06	464.39	484.14	486.29

**Fig. 4:**
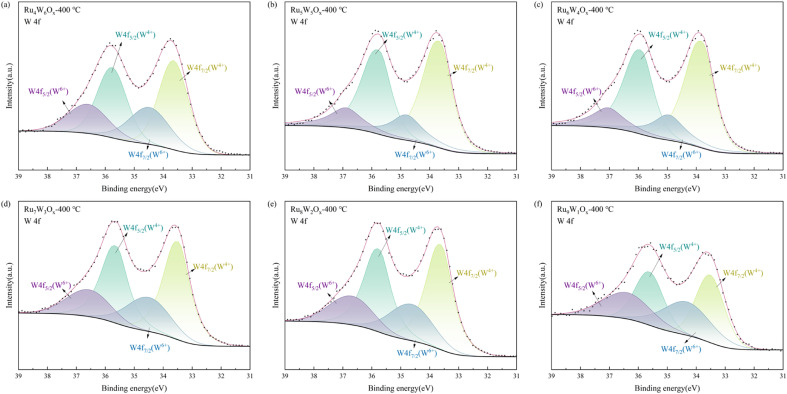
W 4f XPS spectra of (Ru–W)O_*x*_ catalysts (a) Ru_4_W_6_O_*x*_-400 °C, (b) Ru_5_W_5_O_*x*_-400 °C, (c) Ru_6_W_4_O_*x*_-400 °C, (d) Ru_7_W_3_O_*x*_-400 °C, (e) Ru_8_W_2_O_*x*_-400 °C, (f) Ru_9_W_1_O_*x*_-400 °C.

The W 4f spectra (Fig. 4 and Table 3) can be deconvoluted into two pairs of peaks: those located at 33–34 eV, 34–35 eV, 35–36 eV, and 36–37 eV correspond to the oxidation states of W^4+^ (W4f_7/2_), W^6+^ (W 4f_7/2_), W^4+^ (W4f_5/2_), and W^6+^ (W 4f_7/2_), respectively. Notably, the XPS W 4f spectra of Ru_7_W_3_O_*x*_-400 °C, Ru_6_W_4_O_*x*_-400 °C and Ru_5_W_5_O_*x*_-400 °C show higher fractions of W^4+^. This indicates that these catalysts contain more structural defects,^33^ which facilitate electron transfer. Additionally, the binding energies of the W peak for Ru_7_W_3_O_*x*_-400 °C and Ru_6_W_4_O_*x*_-400 °C are relatively higher, suggesting the electron transfer from W to Ru through Ru–O–W interfacial bonds.^32,34^

**Table 3:** The binding energy and proportion of W 4f peaks

Catalysts	W 4f_7/2_ (W^4+^)		W 4f_5/2_ (W^4+^)		W 4f_7/2_ (W^6+^)		W 4f_5/2_ (W^6+^)	
	Binding energy (eV)	Proportion (%)	Binding energy (eV)	Proportion (%)	Binding energy (eV)	Proportion (%)	Binding energy (eV)	Proportion (%)
Ru_9_W_1_O_*x*_-400 °C	33.52	27.13	35.63	27.16	34.33	22.84	36.40	22.86
Ru_8_W_2_O_*x*_-400 °C	33.62	33.04	35.76	33.08	34.62	16.93	36.69	16.95
Ru_7_W_3_O_*x*_-400 °C	33.74	35.36	35.87	35.40	34.66	14.61	36.76	14.62
Ru_6_W_4_O_*x*_-400 °C	33.81	37.22	35.92	37.26	34.95	12.72	37.04	12.80
Ru_5_W_5_O_*x*_-400 °C	33.75	35.37	35.87	34.39	34.53	15.11	36.60	15.13
Ru_4_W_6_O_*x*_-400 °C	33.64	31.85	35.77	31.88	34.48	18.12	36.60	18.14

**Fig. 5:**
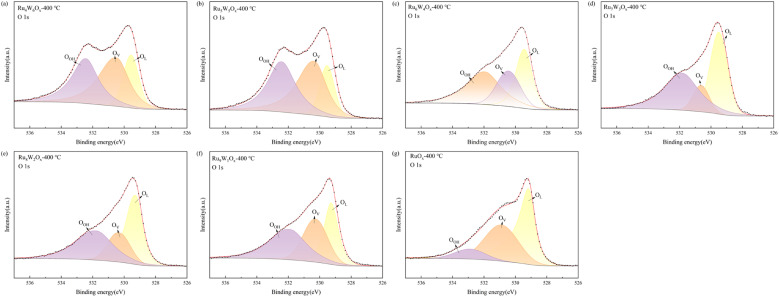
O 1s XPS spectra of (Ru–W)O_*x*_ catalysts (a) Ru_4_W_6_O_*x*_-400 °C, (b) Ru_5_W_5_O_*x*_-400 °C, (c) Ru_6_W_4_O_*x*_-400 °C, (d) Ru_7_W_3_O_*x*_-400 °C, (e) Ru_8_W_2_O_*x*_-400 °C, (f) Ru_9_W_1_O_*x*_-400 °C, (g) RuO_*x*_-400 °C.

The O 1s spectra (Fig. 5 and Table 4) shows a peak at approximately 531–532 eV, corresponding to surface hydroxyl groups (O_OH_). Among the catalysts, RuO_*x*_ exhibits the lowest –OH proportion at just 9.25%, whereas the (Ru–W)O_*x*_ mixed catalysts show a significant increase in –OH content. In particular, Ru_7_W_3_O_*x*_-400 °C and Ru_6_W_4_O_*x*_-400 °C exhibit –OH proportions of 43.72% and 40.54%, respectively, indicating a higher prevalence of surface hydroxyl groups on these mixed catalysts. The binding energy at approximately 530 eV corresponds to oxygen vacancies (O_V_) within the catalyst structure, while the binding energy near 529 eV is attributed to lattice oxygen (O_L_), typically indicative of metal oxide structures. A high proportion of lattice oxygen suggests the presence of more metal oxide structures in the catalyst. In the OER process, surface hydroxyl (O_OH_) groups often serve as critical active sites, as they promote the dissociation of water molecules and facilitate oxygen formation.^8^ These results highlight the enhanced surface properties and catalytic activity of Ru_7_W_3_O_*x*_-400 °C and Ru_6_W_4_O_*x*_-400 °C for the OER.

**Table 4:** The binding energy and proportion of O 1s peaks

Catalysts	O_OH_		O_V_		O_L_	
	Binding energy (eV)	Proportion (%)	Binding energy (eV)	Proportion (%)	Binding energy (eV)	Proportion (%)
RuO_*x*_-400 °C	532.88	9.25	530.89	38.93	529.16	51.83
Ru_9_W_1_O_*x*_-400 °C	531.90	37.23	530.27	32.06	529.27	30.72
Ru_8_W_2_O_*x*_-400 °C	531.76	38.07	530.31	21.56	529.26	40.37
Ru_7_W_3_O_*x*_-400 °C	531.83	43.72	530.63	13.23	529.50	43.05
Ru_6_W_4_O_*x*_-400 °C	531.96	40.54	530.44	26.96	529.41	32.50
Ru_5_W_5_O_*x*_-400 °C	532.42	38.88	530.35	42.17	529.53	18.96
Ru_4_W_6_O_*x*_-400 °C	532.44	36.22	530.48	41.84	529.56	21.94

### 3.2 OER performance

**Fig. 6:**
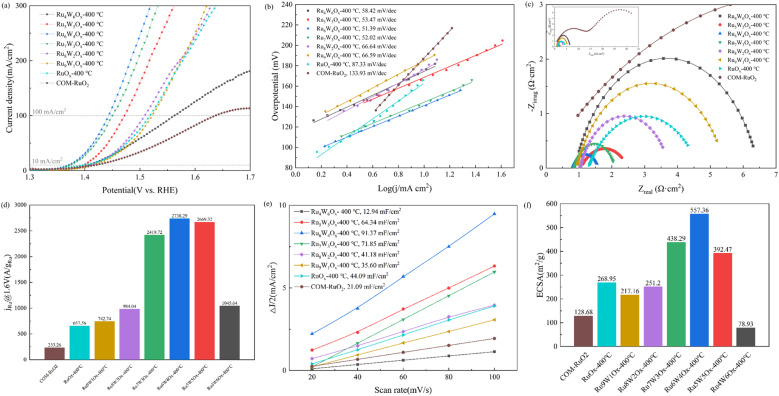
OER performance of catalysts in 0.5 M H_2_SO_4_ (a) polarization curves of catalysts in 0.5 M H_2_SO_4_ (90% IR correction), (b) fitted Tafel plots of catalysts, (c) EIS diagrams of catalysts under 1.45 V (Nyquist plots), (d) mass activity of catalysts, (e) *C*_dl_ plots derived from CV curves, (f) ECSA's calculation results.

The iR-corrected (90% compensation) LSV curves (Fig. 6a) demonstrate that all the synthesized (Ru–W)O_*x*_ catalysts exhibit superior OER performance compared to COM-RuO_2_. At the same current density, the overpotential of (Ru–W)O_*x*_ catalysts is significantly lower than that of COM-RuO_2_ (187.59 mV@10 mA cm^−2^ and 406.57 mV@100 mA cm^−2^). As the W content increases, the overpotential of (Ru–W)O_*x*_ catalysts initially decreases and then increases, reaching the lowest values with Ru_6_W_4_O_*x*_ (140.32 mV@10 mA cm^−2^ and 213.16 mV@100 mA cm^−2^), which are much lower than those of COM-RuO_2_. This suggests that the appropriate W doping ratio can effectively enhance the catalytic activity of the OER. However, when the W content exceeds 60 at%, the overpotential sharply increases, likely due to excessive W partially substituting or covering Ru active sites, leading to unfavorable changes in the crystal structure. The oxidation peak observed at approximately 1.65 V for COM-RuO_2_ can be attributed to the oxidation of residual low-valence Ru species in the commercial catalyst. Due to the limited amount of oxidizable Ru^3+^, when these low-valent Ru species are fully oxidized, the current contribution of the reaction reaches saturation, manifested as the current density no longer increases with voltage.

The Tafel slopes of Ru_6_W_4_O_*x*_-400 °C, Ru_7_W_3_O_*x*_-400 °C, and Ru_5_W_5_O_*x*_-400 °C are the lowest among all catalysts, at 51.39 mV dec^−1^, 52.02 mV dec^−1^, and 53.47 mV dec^−1^, respectively (Fig. 6b). This indicates that these catalysts exhibit the fastest reaction kinetics, allowing high current density at lower overpotential and making the OER process more efficient. The optimal W doping level (30–50 at%) improves the electronic structure and reaction kinetics, significantly enhancing the OER performance of Ru-based catalysts. In contrast, COM-RuO_2_ shows the highest Tafel slope (133.93 mV dec^−1^), reflecting the poorest OER performance and underscoring the advantages of W-doped catalysts in OER reactions.

The Nyquist plots (Fig. 6c) show the impedance characteristics of (Ru–W)O_*x*_ catalysts with different doping ratios compared to COM-RuO_2_. The Nyquist plot of COM-RuO_2_ displays two semicircles, indicating two main limiting steps in the OER: a high-frequency process associated with charge transfer and a low-frequency process related to diffusion or interfacial resistance. These results suggest that the catalytic performance of COM-RuO_2_ is limited by its high charge transfer resistance and poor gas or ion transport properties. Ru_4_W_6_O_*x*_-400 °C exhibits the highest charge transfer resistance, likely due to the excessive W doping reducing the electrical conductivity of the catalyst and hindering electron transfer. In contrast, Ru_6_W_4_O_*x*_-400 °C and Ru_7_W_3_O_*x*_-400 °C show much higher charge transfer efficiency, with optimal W doping minimizing activation polarization and improving electron conductivity. This optimization of the electronic structure contributes to enhanced OER performance.

As shown in Fig. 6d, Ru_6_W_4_O_*x*_-400 °C exhibits an outstanding mass activity of 2738.29 A g_Ru_^−1^ at 1.6 V, which is 4 times that of RuO_*x*_ (657.56 A g_Ru_^−1^) and 12 times that of COM-RuO_2_ (233.26 A g_Ru_^−1^).

The electrochemical double-layer capacitance (*C*_dl_) was tested to calculate the ECSA (Fig. 6e and f). Ru_6_W_4_O_*x*_-400 °C exhibited the largest ECSA of 557.36 m^2^ g^−1^, which is 2.07 times that of RuO_*x*_ and 4.33 times that of COM-RuO_2_. This indicates that Ru_6_W_4_O_*x*_-400 °C provides the highest number of active sites for OER reactant adsorption, further validating its superior catalytic activity. The low ECSA of COM-RuO_2_ leads to the difficulty of OER reactant adsorption and rapid desorption of O_2_ on the surface, further inhibiting current growth, which also accounts for the phenomenon of current stagnation.

**Fig. 7:**
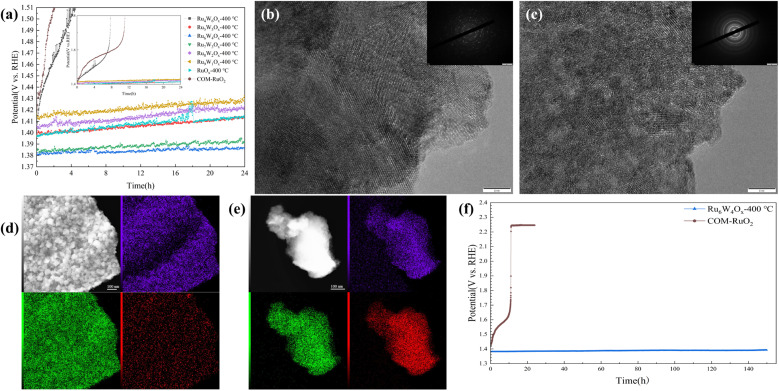
Chronopotentiogram and characterization after acidic OER (a) chronopotentiogram of different catalysts at 10 mA cm^−2^, (b) HRTEM images and SAED patterns of Ru_4_W_6_O_*x*_-400 °C after OER, (c) HRTEM images and SAED patterns of Ru_6_W_4_O_*x*_-400 °C after OER, (d) EDX elemental mapping of Ru_4_W_6_O_*x*_-400 °C after OER, (e) EDX elemental mapping of Ru_6_W_4_O_*x*_-400 °C after OER, (f) chronopotentiogram of Ru_6_W_4_O_*x*_-400 °C and COM-RuO_2_ over an extended duration.

From a practical application perspective, stability in acidic media is a critical parameter for evaluating OER electrocatalysts. The chronoamperometric results (Fig. 7a) reveal the stability of various (Ru–W)O_*x*_ catalysts compared to COM-RuO_2_ during the OER. COM-RuO_2_ shows a significant performance decline within the first hour, indicating rapid deactivation during prolonged OER reactions. Impressively, Ru_6_W_4_O_*x*_-400 °C exhibits minimal activity decay over 24 hours, with the working electrode potential increasing by only about 3 mV. This demonstrates the high structural stability of the Ru–W doped heterostructure at this ratio. The stability enhancement is likely due to the formation of strong Ru–O–W bonds, which reduce Ru particle aggregation and dissolution. Ru particles are prone to dissolution or reconstruction under high potentials during OER. However, appropriate W doping mitigates this process, preserving the catalyst's original nanostructure and enhancing its long-term stability. The Ru_4_W_6_O_*x*_-400 °C, on the other hand, shows a significantly higher initial electrode potential compared to other (Ru–W)O_*x*_ catalysts due to its poor OER activity, consistent with the LSV and EIS results. Its electrode potential sharply increases after just 0.5 hours of testing, indicating rapid deactivation. This may be attributed to excessive W doping, which distorts Ru's crystal structure or forms unstable oxide phases that are prone to reconstruction or dissolution during the reaction, leading to the loss of active sites and catalyst surface passivation. The TEM images and SAED patterns of Ru_6_W_4_O_*x*_-400 °C and Ru_4_W_6_O_*x*_-400 °C after OER (Fig. 7b and c), demonstrate that Ru_6_W_4_O_*x*_-400 °C maintains structural stability under OER conditions, while the structure and morphology of Ru_4_W_6_O_*x*_-400 °C are significantly different from those before OER. According to the elemental mapping (Fig. 7d and e and Table S1), Ru_4_W_6_O_*x*_-400 °C exhibits a significant decrease in W content, while Ru_6_W_4_O_*x*_-400 °C shows little change.

Based on the combined OER activity and stability results, Ru_6_W_4_O_*x*_-400 °C demonstrates a robust balance of activity and stability. Its synergistic structure and strong Ru–O–W bonds provide durable functional active sites, showcasing high potential for practical applications. Consequently, its stability was assessed over an extended duration at a constant current density of 10 mA cm^−2^ (Fig. 7f). Ru_6_W_4_O_*x*_-400 °C demonstrated exceptional durability, exhibiting a negligible potential increase of 12 mV over 150 h of continuous operation. In contrast, COM-RuO_2_ underwent rapid deactivation within 10 h.

### 3.3. PEMWE device performance

**Fig. 8:**
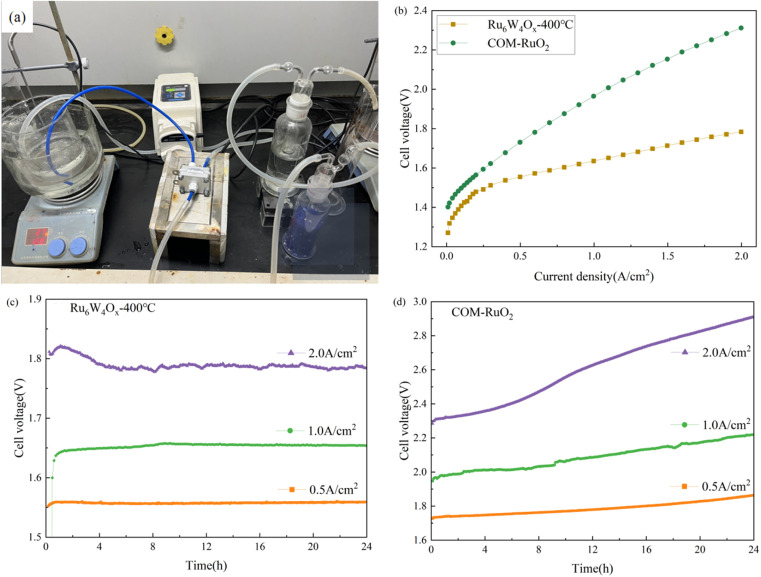
PEMWE device performance at 80 °C and ambient pressure (a) photograph of the PEMWE device, (b) *I*–*V* curves of PEMWE electrolyzers using Ru_6_W_4_O_*x*_-400 °C or COM-RuO_2_ as anodic catalyst and commercial Pt/C as cathodic catalyst, at room temperature and ambient pressure. No cell voltages were iR compensated, (c) chronopotentiogram of Ru_6_W_4_O_*x*_-400 °C at 0.5 A cm^−2^, 1 A cm^−2^, and 2 A cm^−2^, (d) chronopotentiogram testing of COM-RuO_2_ at 0.5 A cm^−2^, 1 A cm^−2^, and 2 A cm^−2^.

Finally, a PEM water electrolyzer (PEMWE) was constructed to evaluate the practical application potential of the Ru_6_W_4_O_*x*_-400 °C catalyst for water electrolysis as shown in Fig. 8a. The electrolyzer utilized Ru_6_W_4_O_*x*_-400 °C as the anode catalyst, commercial 60 wt% Pt/C (purchased from Suzhou Sinero Co., Ltd) as the cathode catalyst, and a proton exchange membrane (Nafion 115). Catalyst coated on membrane (CCM) is employed to prepare 2 × 2 cm^2^ active area membrane electrode assembly (MEA). For comparison, another electrolyzer was assembled using COM-RuO_2_ as the anode catalyst, with all other conditions kept identical.

The polarization curves (Fig. 8b) show that the electrolyzer using the self-synthesized Ru_6_W_4_O_*x*_-400 °C catalyst exhibits significantly lower voltages across the entire current density range compared to the COM-RuO_2_-based system. This indicates that the self-synthesized catalyst achieves lower energy consumption at the same current density.

Due to laboratory safety regulations, chronoamperometric tests were conducted at current densities of 0.5 A cm^−2^, 1 A cm^−2^, and 2 A cm^−2^ for 24 hours (Fig. 8c and d) to assess the stability of the catalysts. The electrochemical stability of the electrolyzers varied significantly between the self-synthesized Ru_6_W_4_O_*x*_-400 °C catalyst and COM-RuO_2_, especially at high current densities. The Ru_6_W_4_O_*x*_-400 °C-based electrolyzer demonstrated stable voltage throughout the 24 hours test, maintaining a consistent voltage of approximately 1.78 V at 2 A cm^−2^ with minimal fluctuations, indicating excellent stability. In contrast, the COM-RuO_2_-based electrolyzer exhibited significantly poorer electrochemical stability under the same conditions. At 2 A cm^−2^, its initial voltage of 2.2 V increased to nearly 2.8 V over the 24 hours test, showing a clear trend of voltage degradation.

Additionally, hydrogen production efficiency, electrolysis efficiency, and energy consumption values for Ru_6_W_4_O_*x*_-400 °C at a current density of 2 A cm^−2^ and a working temperature of 80 °C were calculated and are presented in Table 5. The electrolyzer with Ru_6_W_4_O_*x*_-400 °C as the anode catalyst achieved a lower cell voltage of 1.784 V and hydrogen production energy consumption of 4.34 kWh m^−3^ H_2_, with an electrolysis efficiency of 81.72%.

**Table 5:** Performance of Ru_6_W_4_O_*x*_-400 °C-based electrolyzer at 80 °C and 2 A cm^−2^

The anode catalyst	Potential (V)	Actual H_2_ production (L)	Theoretical H_2_ production (L)	H_2_ production efficiency (%)	Electrolytic efficiency (%)	Energy consumption (kWh m^−3^ H_2_)
Ru_6_W_4_O_*x*_-400 °C	1.784	3.288	3.343	98.34	81.72	4.34

Representative Ru-based catalysts for PEM electrolyzer were systematically compared in Table 6. The Ru_6_W_4_O_*x*_-400 °C catalyst developed in this work demonstrates significant advantages, including a remarkably low overpotential of 140.32 mV@10 mA cm^−2^, an ultrahigh mass activity of 2738.2 A g_Ru_^−1^ at 1.6 V, exceptional stability with only a 3 mV increase in *η*_10_ over 24 hours. Notably, it achieves a competitive operating voltage of 1.784 V at 2 A cm^−2^, and maintains no performance decay after 24 hours under practical high-current-density conditions. These metrics collectively highlight its potential for efficient water electrolysis systems.

**Table 6:** Comparison of representative Ru-based electrocatalysts performance

Catalyst	Electrolyte	In a three-electrode system				In a PEMWE device		Ref.
		*η*_10_ (mV)	Tafel slope (mV dec^−1^)	Mass activity	Stability	Voltage (V)	Stability	
Ru_6_W_4_O_*x*_-400 °C	0.5 M H_2_SO_4_	140.32	51.39	2738.2 A g_Ru_^−1^@1.6 V	*η*_10_ increased by 3 mV in 24 h	1.784 V@2 A cm^−2^	No decay was observed after 24 h@2 A cm^−2^	This work
RuO_2_ NSs	0.5 M H_2_SO_4_	199	38.2	0.52 A mg_Ru_^−1^@1.46 V	*η*_10_ increased by 32 mV in 6 h	1.65 V@0.93 A cm^−2^	—	35
Ru_1_-Pt_3_Cu	0.1 M HClO_4_	220	—	779 A g_Ru_^−1^_+ Pt_@1.51 V	*η*_10_ increased by 2.1% in 28 h	—	—	16
Cu-doped RuO_2_	0.5 M H_2_SO_4_	188	43.96	—	*η*_10_ increased by 83 mV in 8 h	—	—	30
np-UHEA12	0.5 M H_2_SO_4_	258	84.2	—	—	1.53 V@10 mA cm^−2^	Voltage increased by 100 mV in 40 000 s@10 mA cm^−2^	17
RuCu NSs/C −350 °C	0.5 M H_2_SO_4_	236	40.4	—	—	1.49 V@10 mA cm^−2^	Stable for 20.5 h@5 mA cm^−2^	18
Core–shell Ru@IrO_*x*_	0.5 M H_2_SO_4_	282	69.1	644.8 A g_Ru_^−1^@1.56 V	Activity declined to nearly 90% in 24 h@10 mA cm^−2^	—	—	36
RuO_2_ NWs	0.5 M H_2_SO_4_	234	—	—	45 mV overpotential change in 20 h@5 mA cm^−2^	1.50 V@10 mA cm^−2^	Voltage increased by 30 mV in 10 h@5 mA cm^−2^	37
Nb_0.1_Ru_0.9_O_2_	0.5 M H_2_SO_4_	204	47.9	150.5 A g_Ru_^−1^@1.47 V	No obvious decay was observed in 300 h@200 mA cm^−2^	1.69 V@1 A cm^−2^	Survived for 100 h@300 mA cm^−2^	15
Co_0.11_Ru_0.89_O_2−*δ*_ (350)	0.5 M H_2_SO_4_	169	49	—	*η*_10_ increased by 15 mV after 10 000 cycles	—	—	19
S-RuFeO_*x*_	0.1 M HClO_4_	187	40	1.18 A mg_Ru_^−1^@1.42 V	*η*_10_ increased by 20 mV in 10 h	—	—	20
W-IrRu_3_O_*x*_	0.5 M H_2_SO_4_	249	64	3.2 A mg_Ir+Ru_^−1^@1.53 V	*η*_10_ increased from 231 to ∼300 mV in the first ∼30 h	1.66 V@1 A cm^−2^ and 1.82 V@2 A cm^−2^	Stable for 24 h@1.5 A cm^−2^	21
Mn-RuO_2_	0.5 M H_2_SO_4_	158	42.94	596.38 A g_Ru_^−1^@1.5 V	Stable for 10 h@10 mA cm^−2^	—	—	22
Cr_0.6_Ru_0.4_O_2_ (550)	0.5 M H_2_SO_4_	178	58	229 A g_Ru_^−1^@1.5 V	*η*_10_ increased by 11 mV after 10 000 cycles	—	—	23
a-RuTe_2_ PNRs	0.5 M H_2_SO_4_	245 mV	—	—	—	1.52 V@10 mA cm^−2^	Stable for 24 h@10 mA cm^−2^	24
Ru/RuS_2_	0.5 M H_2_SO_4_	201	47.2	—	Stable for 24 h@10 mA cm^−2^	1.501 V@10 mA cm^−2^	Slightly decayed in 10 h@ 10 mA cm^−2^	38
Y_2_Ru_2_O_7−*3*_	0.1 M HClO_4_	190	55	—	Decreased by 0.17 mA cm^−2^ after 10 000 cycles	—	—	25
M-RuO_2_	0.5 M H_2_SO_4_	230	—	—	Stable for 4 h@1.53 V	1.9V@1 A cm^−2^	Voltage didn't change significantly in 100 h	26
Y_1.7_Sr_0.3_Ru_2_O_7_	0.5 M H_2_SO_4_	264	44.8	1018 A g_Ru_^−1^@1.53 V	Stable for 28 h@10 mA cm^−2^	—	—	27
Nd_2_Ru_2_O_7_	0.1 M HClO_4_	210	58.48	—	Stable for 6 h@1 mA cm^−2^	—	—	28
V-Ru_*x*_Mn_1−*x*_O_2_ NWs	0.5 M H_2_SO_4_	200.0	48.97	—	Stable for 600 h@10 mA cm^−2^	1.48 V@10 mA cm^−2^ &1.61 V@50 mA cm^−2^	Stable for >30 h@500 mA cm^−2^	29

## 4 Conclusion

A cost-effective anode catalyst for hydrogen production *via* PEM water electrolysis was developed using a co-doping strategy with Ru and W. The incorporation of tungsten not only reduces the reliance on expensive noble metals but also improves the catalyst's activity and stability through its synergistic interaction with ruthenium. Using a simple and efficient preparation process, tungsten was introduced at an optimal ratio, which increased the number of active sites and facilitated precise control over the catalyst's nanoscale structure, significantly enhancing its electrochemical performance for the oxygen evolution reaction. The optimized Ru_6_W_4_O_*x*_-400 °C catalyst demonstrated remarkable catalytic efficiency and durability, achieving an overpotential of just 140.32 mV at 10 mA cm^−2^ with only a 12 mV in overpotential increase after 150 hours of continuous electrolysis. Additionally, a PEMWE employing Ru_6_W_4_O_*x*_-400 °C as the anode catalyst delivered outstanding performance, achieving a cell voltage as low as 1.784 V at a current density of 2 A cm^−2^, an energy consumption of 4.34 kWh m^−3^ H_2_, and an electrolysis efficiency of 81.72%. As summarized in Table 6, the Ru_6_W_4_O_*x*_-400 °C catalyst exhibits clear advantages over other catalysts. This work contributes to advancing the development of low-cost, high-efficiency water electrolysis technology for hydrogen production.

## Conflicts of interest

There are no conflicts to declare.

## Supplementary Material

RA-015-D5RA05145B-s001

## Data Availability

The data that supports the findings of this study are available from the corresponding authors upon reasonable request. Supplementary information: experimental details, supplementary tables and the effect of annealing temperature on catalyst performance. See DOI: https://doi.org/10.1039/d5ra05145b.
