# Supplementary material for: Ruthenium-based electrocatalyst for efficient acidic water oxidation in PEM water electrolysis for H2 production
Source: RSC Adv. 2025 Sep 23;15(42):34991–5002. doi: 10.1039/d5ra05145b (PMC12455425; doi:10.1039/d5ra05145b)
Supplement: RA-015-D5RA05145B-s001 [file RA-015-D5RA05145B-s001.pdf]

Supplementary Information (SI) for RSC Advances.
This journal is © The Royal Society of Chemistry 2025

Supplementary Information for

# Ruthenium-based electrocatalyst for efficient acidic water oxidation in PEM water electrolysis for $H_2$ production

**Yinan Tao[a], Ruilin Zhang[c], Junyan Chen[a], Wubin Weng[a,b], Yong He[a,b], Zhihua Wang[a,b,*]**

[a] State Key Laboratory of Clean Energy Utilization, Zhejiang University, Hangzhou 310027, P. R. China

[b]Qingshanhu Energy Research Center, Zhejiang University, Hangzhou, 311300, P. R. China

[c]Hoymiles Power Electronics Inc., Hangzhou, 310015, P. R. China

Corresponding author: wangzh@zju.edu.cn

## Experimental Details

## Materials and Chemicals

Ruthenium (III) chloride trihydrate ($RuCl_3 \cdot 3H_2O$), Tungsten (VI) chloride ($WCl_6$), and the commercial $RuO_2$ catalyst were purchased from Shanghai Macklin Biochemical Co.,Ltd., and used without further purification. Ethylenediaminetetraacetic acid (EDTA, $C_{10}H_{16}N_2O_8$), citric acid ($C_6H_8O_7$), and deionized water were sourced from Aladdin Chemistry Co., Ltd. Isopropanol (>99.5%), ethanol (>97%), perchloric acid ($HClO_4$), concentrated sulfuric acid ($H_2SO_4$) were acquired from Sinopharm Chemical Reagent Co., Ltd. Nafion solution (5 wt.%) was supplied by DuPont Co., Ltd.

## Materials Characterization

The morphological characteristics and elemental distribution of the catalysts were investigated using a field-emission transmission electron microscope (FE-TEM, FEI Tecnai G2 F20 S-TWIN) equipped with energy-dispersive X-ray spectroscopy (EDX) for spatially resolved elemental mapping. X-ray photoelectron spectroscopy (XPS, Thermo Scientific K-Alpha) was used to analyze the surface elemental composition and oxidation states of the catalysts. Crystallographic analysis was performed through X-ray diffraction (XRD, X-pert Powder) with a scan range of $2\theta$=20-80° at 0.02° step resolution to determine phase composition and lattice parameters.

**Calculation of Tafel slope**.

The Tafel slopes were calculated using polarization curves at small overpotentials according to the Tafel equation:

$$\eta = a + b \log|j| \quad (1)$$

where η is the overpotential, j represents the current density, a represents a constant, and b is the Tafel slope.

**Calculation of the double-layer capacitance ($C_{dl}$) and the electrochemically active surface area (ECSA)**.

The $C_{dl}$ value was obtained by linear fitting of the geometric current density difference at the middle position of CV curve in the non-Faraday region with the scanning rate. Half of the slope of the fitted line is the double layer capacitance (Cdl) of the catalyst.

The ECSA was evaluated from the double-layer capacitance from the equation below:

$$\text{ECSA}=\frac{C_{dl}}{C_s m_{loading}} \quad (2)$$

$C_s$ is the sample's specific capacitance or the capacitance of an atomically smooth planar surface of the material per unit under identical electrolyte conditions, and is usually assumed as 0.035 $mF/cm^2$ in $H_2SO_4$ electrolyte; $m_{loading}$ is the mass of the load catalyst.

**Calculation of Mass Activity**

The mass activity ($j_m$) of the catalysts was calculated using the equation below:

$$j_{Ru} = \frac{j}{m_{loading} \times Ru(wt.\%)} \quad (3)$$

Where Ru(wt.%) is the mass fraction of Ru in the catalyst calculated from EDS analysis, j is the geometric current density.

**Calculation of hydrogen production efficiency, energy consumption value and electrolysis efficiency**

The hydrogen production efficiency η, energy consumption value w and electrolysis

efficiency $\eta_e$ of the self-made membrane electrode are calculated by formula (4), formula (7) and formula (8).

$$\eta = \frac{V_{exp}}{V_{theo}} \tag{4}$$

$$V_{theo} = \frac{It}{2F} \times V_m \tag{5}$$

Where $V_{exp}$ is the actual hydrogen production volume (L), $V_{theo}$ is the theoretical hydrogen production volume (L), I is the applied constant current, t is the collection time, F is the Faraday constant (96485 C/mol), and $V_m$ is the gas molar volume (22.4 L/mol).

$$W = \frac{I \cdot \int_0^t E dt}{1000} \tag{6}$$

$$w = \frac{W}{V_{exp}} \tag{7}$$

W is the electric energy (kJ) consumed during the collection time, which can be obtained by integrating a certain interval within the curve obtained by the timing amperometric method. The energy consumption per unit volume of hydrogen production (w, $kWh/m^3H_2$) is the ratio of the actual electric energy consumed to the actual hydrogen production.

$$\eta_e = \frac{Q_{H2}}{w} \times 100\% \tag{8}$$

The electrolysis efficiency $\eta_e$ is the ratio of the heat release per unit volume of $H_2$ to the energy consumption required to produce $H_2$. $Q_{H2}$ is the calorific value of heat release per unit volume of $H_2$ (12797.3549 $kJ/m^3$).

## Supplementary Figures and Tables

**Table S1 Elemental composition of $Ru_6W_4O_x$-400 °C and $Ru_4W_6O_x$-400 °C after OER**

| Catalysts | Ru (at%) | W (at%) | O (at%) | W/Ru |
|---|---|---|---|---|
| $Ru_6W_4O_x$-400 °C | 20.45 | 12.54 | 67.01 | 0.61 |
| $Ru_4W_6O_x$-400 °C | 37.84 | 0.35 | 61.81 | 0.0092 |

**Effect of preparation temperature on OER performance of (Ru-W)$O_x$ catalyst**.

In addition to W content, we also studied the effect of annealing temperature on catalyst performance.

The electrochemical activity curves for the OER of catalysts prepared at different temperatures are shown in Fig. S1. The polarization curves (90% iR-corrected) reveal that for $Ru_6W_4Ox$ catalysts, those calcined at 350 °C, 400 °C, 450 °C, and 500 °C exhibited overpotentials of 130.71 mV, 140.32 mV, 186.46 mV, and 192.08 mV at a current density of 10 mA/cm², respectively. This indicates that catalysts synthesized at lower temperatures demonstrate lower OER overpotential. However, as current density increases, the overpotential of $Ru_6W_4O_x$-350 °C rises more rapidly, showing inferior performance compared to $Ru_6W_4O_x$-400 °C at high current densities. At 100 mA/cm², the overpotential of $Ru_6W_4O_x$-400 °C reaches 213.16 mV while that of $Ru_6W_4O_x$-350 °C reaches 233.53 mV. The trend in the curves further suggests that the overpotential rise rate of $Ru_6W_4O_x$-350 °C accelerates beyond $Ru_6W_4O_x$-450 °C as current density increases. Notably, when the preparation temperature rises to 500 °C, the current density decreases, indicating that excessively high temperatures may negatively impact catalyst performance. Thus, although the catalyst prepared at 350 °C low temperature performs better under low potential, its current density is not as high as $Ru_6W_4O_x$-400 °C and $Ru_6W_4O_x$-450 °C under high potential. In general, $Ru_6W_4O_x$-400 °C has better catalytic effect under high current application conditions.

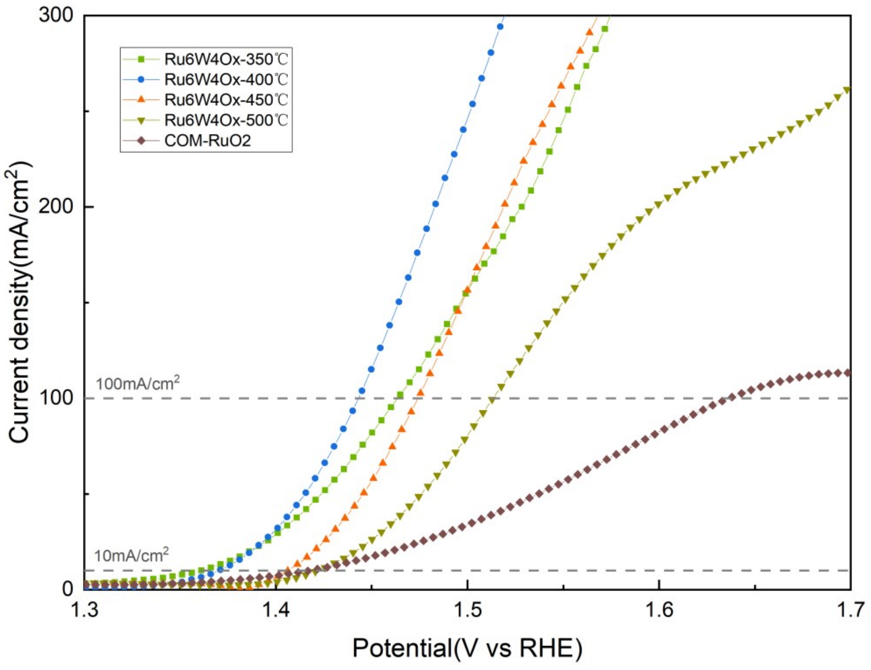


**Fig. S1 Polarization curves of $Ru_6W_4O_x$ catalysts prepared at different temperatures in 0.5 M $H_2SO_4$ solution (90% iR corrected)**

The Tafel curve revealed that the $Ru_6W_4O_x$ catalyst prepared at 400 °C exhibited optimal electrochemical activity and kinetic characteristics during the OER process. The catalyst not only demonstrated high current density at low overpotential but also showed the smallest Tafel slope (51.39 mV/dec), indicating faster oxygen evolution kinetics.

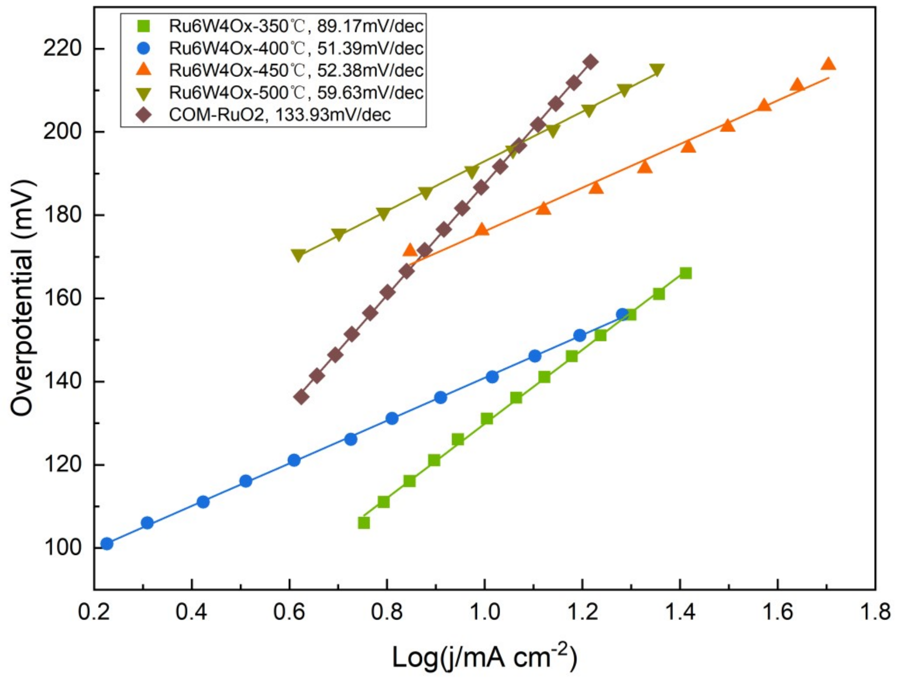

**Fig. S2 Tafel fitting curves of $Ru_6W_4O_x$ catalysts prepared at different temperatures**

In EIS tests, $Ru_6W_4O_x$ catalysts prepared at different temperatures exhibited significant differences in charge transfer resistance ($R_{ct}$) and diffusion resistance, which reflects their electrochemical behavior during the OER. Specifically, the $Ru_6W_4O_x$-400 °C showed the lowest $R_{ct}$, and the smallest first semicircle in its Nyquist plot indicates fast catalytic kinetics. This suggests electrons can be transferred more efficiently from the electrode to reactants, consistent with its excellent performance in LSV tests.

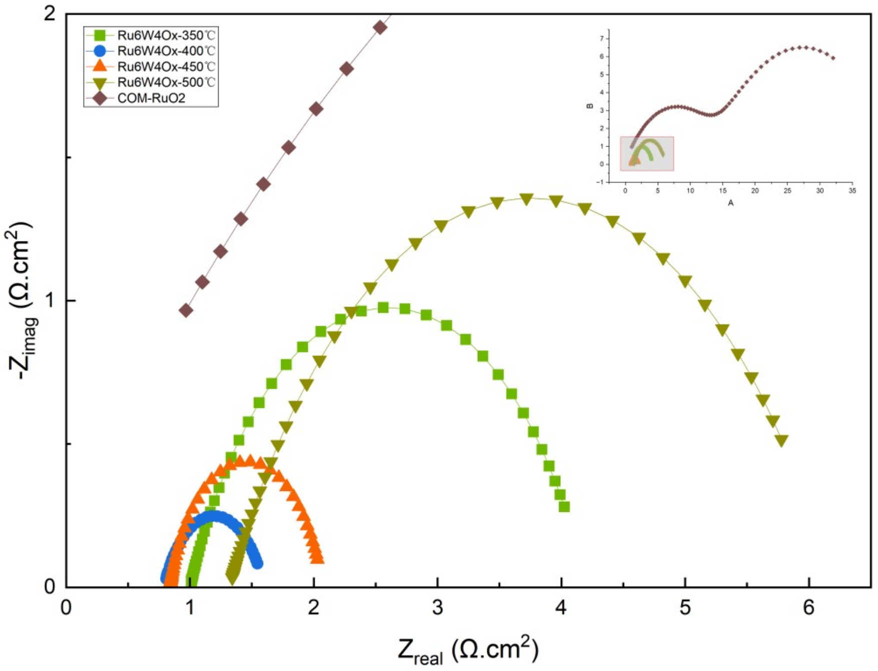


**Fig. S3 Nernst diagram of $Ru_6W_4O_x$ catalysts prepared at different temperatures**

The Ru mass activity of catalysts prepared at different temperatures was measured at an operating voltage of 1.6 V. The results showed that $Ru_6W_4O_x$-400 °C showed the highest mass activity, reaching 2738.29 A/$g_{Ru}$.

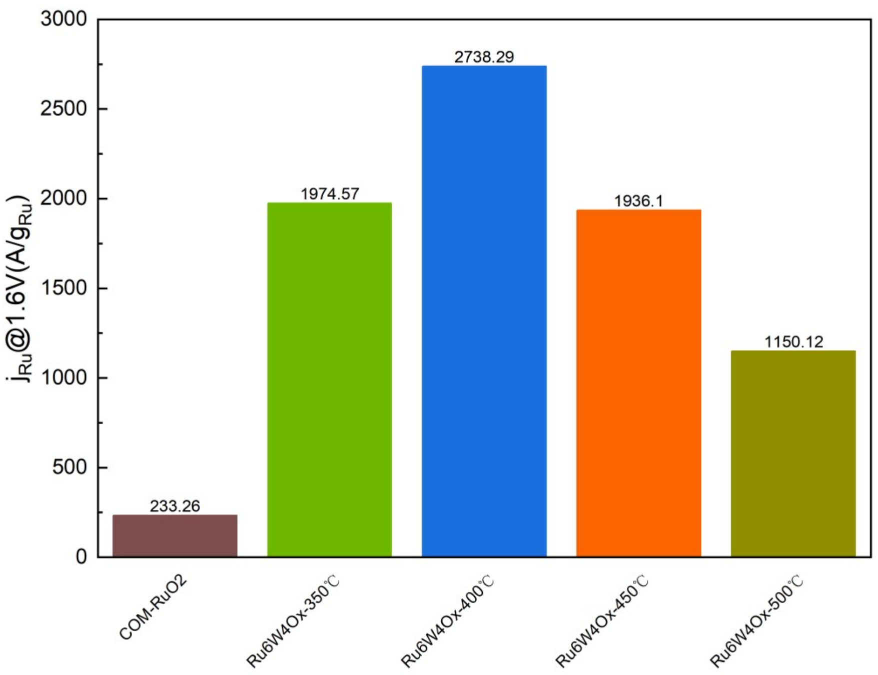


**Fig. S4 Comparison of Ru mass activity of $Ru_6W_4O_x$ catalysts prepared at different temperatures**

As shown in Fig. S5 of the electrochemical double-layer capacitance test results, $Ru_6W_4O_x$-400 °C exhibits the highest $C_{dl}$ value (91.37 mF/cm²), which indicates the largest number of active sites on its surface. This aligns with previous LSV, Tafel, and EIS test results, demonstrating that samples prepared at this temperature possess the most abundant active site distribution and maximum effective surface area. These features provide more electron transfer pathways and reaction sites, thereby enhancing oxygen evolution. Commercial $RuO_2$ shows the lowest $C_{dl}$ value and ECSA, further highlighting the significant advantage of $Ru_6W_4O_x$ materials in terms of active site quantity.

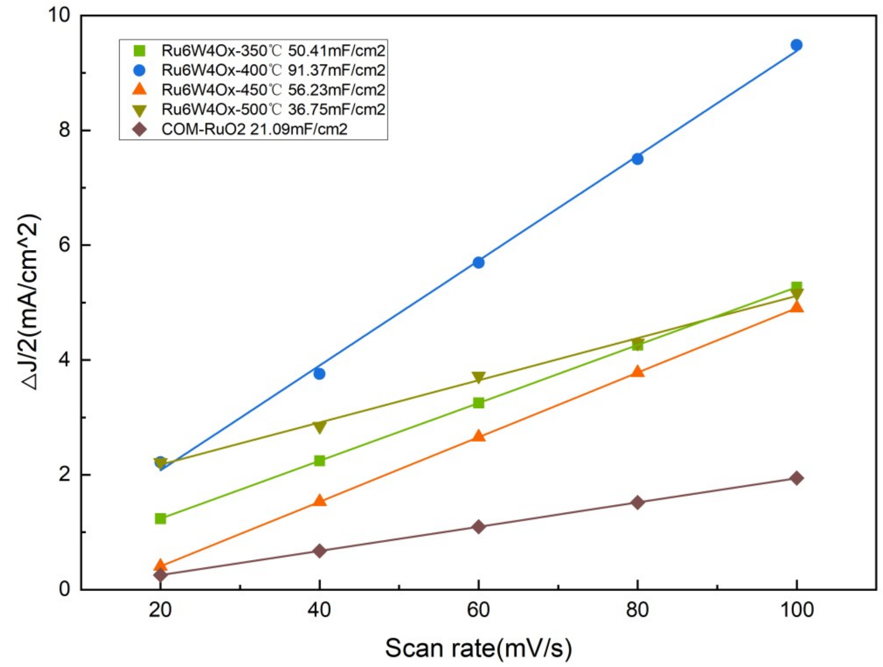


**Fig. S5 Electrochemical double layer capacitance of $Ru_6W_4O_x$ catalysts prepared at different temperatures**

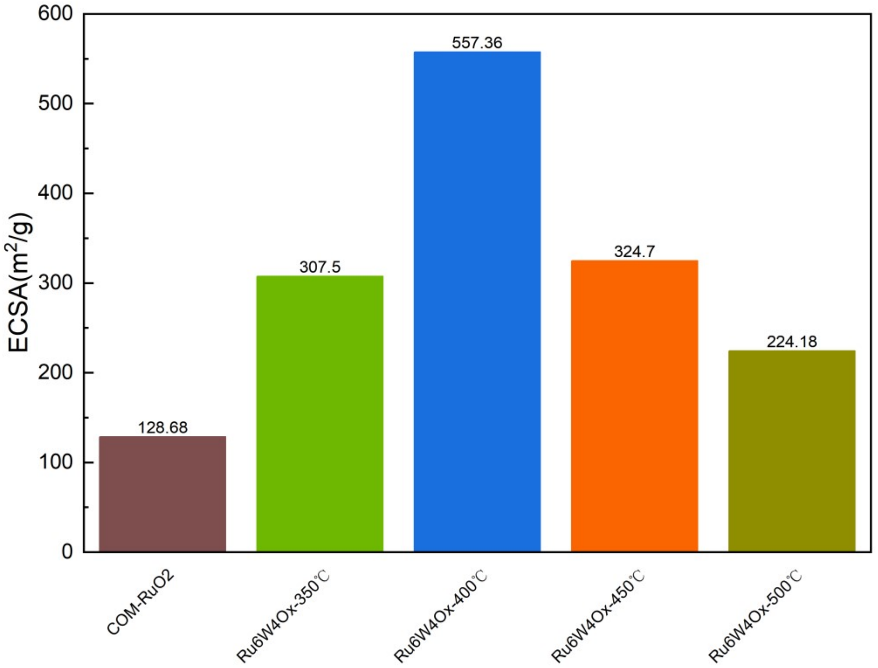


**Fig. S6 Calculation results of electrochemical surface area of $Ru_6W_4O_x$ catalysts prepared at different temperatures**

Under a constant current of 10 mA/cm², the stability of (Ru-W)$O_x$ catalysts prepared at different temperatures was verified and compared using the chronoamperometric method. The catalysts prepared at different temperatures

exhibited significant differences in OER stability, with the 400 °C-prepared catalyst demonstrating the best stability. It maintained a low and stable potential for 24 hours, showing excellent anti-decay capability.

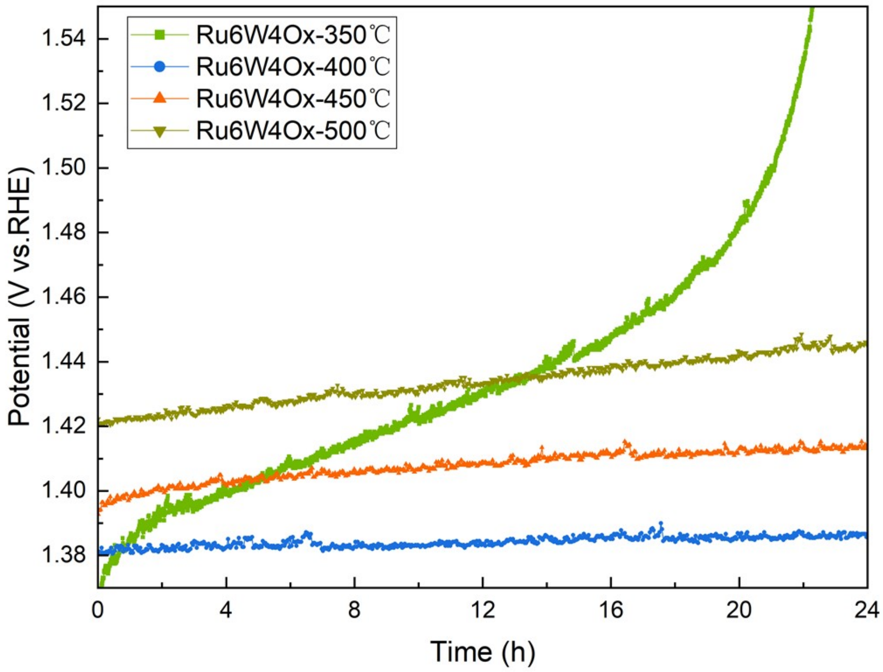


**Fig. S7 Chronopotentiogram of different catalysts in 0.5 M $H_2SO_4$**
